# Cardiovascular and type 2 diabetes morbidity and all-cause mortality among diverse chronic inflammatory disorders

**DOI:** 10.1136/heartjnl-2017-311214

**Published:** 2017-06-10

**Authors:** Alex Dregan, Phil Chowienczyk, Mariam Molokhia

**Affiliations:** 1 Department of Primary Care and Public Health Sciences, King’s College London, London, UK; 2 National Institute for Health Research Biomedical Research Centre, Guy’s and St Thomas NHS Foundation Trust, London, UK; 3 British Foundation Centre, King’s College London, London, UK

**Keywords:** Cardiac risk factors and prevention, Coronary artery disease, Stroke, Systemic inflammatory diseases, Diabetes

## Abstract

**Objectives:**

The present study aimed to assess the relationship between inflammatory disorders with cardiometabolic diseases and mortality within a community-based population.

**Methods:**

The UK Biobank data were used to conduct two investigations: a cross-sectional study to estimate cardiometabolic risk and a prospective cohort study to estimate mortality risk. Binary regression analyses were used to model the association between coronary heart disease, stroke, type 2 diabetes, venous thromboembolism and peripheral artery disease diagnoses with seven inflammatory disorders (eg, rheumatoid arthritis (RA), systemic lupus erythematosus (SLE), psoriasis, ankylosing spondylitis (AS), systemic vasculitis, Crohn’s disease and ulcerative colitis (UC)). Cox proportional hazards was used to estimate all-cause and cardiovascular-related mortality.

**Results:**

About 4% (n=19, 082) of the study population (n=5 02 641) were diagnosed with a chronic inflammatory disorder. The most common inflammatory disorder was psoriasis (n=6286), and the least common was SLE (n=654). SLE showed the strongest association with multiple (relative risk (RR) 6.36, 95% CI 4.37 to 9.25) risk of cardiometabolic diseases, followed by the RA (RR 1.70, 95% CI 1.59 to 1.83), UC (RR 1.69, 95% CI 1.51 to 1.89), AS (RR 1.28, 95% CI 1.09 to 1.52), vasculitis (RR 1.64, 95% CI 1.42–1.90) and psoriasis (RR 1.25, 95% 1.16 to 1.35) disorders. The magnitude of the association was higher among participants prescribed non-steroidal anti-inflammatory drugs or corticosteroids drugs, with multiple cardiometabolic risk being greater within SLE (RR 12.35, 95% CI 7.18 to 21.24), followed by UC (RR 3.81, 95% CI 2.69 to 5.38), Crohn’s disease (RR 3.07, 95% CI 1.85 to 5.11), RA (RR 3.06, 95% CI 2.44 to 3.85), psoriasis (RR 2.36, 95% CI 1.88 to 2.95), AS (RR 2.25, 95% CI 1.48 to 3.41) and vasculitis (RR 1.89, 95% CI 1.28 to 2.79). Similar pattern was observed with respect to the cumulative cardiometabolic risk.

**Conclusion:**

Inflammatory disorders are associated with heightened risk of cardiometabolic events, which may vary by anti-inflammatory therapy and duration. All-cause mortality was also higher among specific inflammatory disorders compared with the absence of inflammatory disorders.

## Introduction

An accumulating body of evidence documents an important role of inflammation in cardiovascular disease (CVD) onset and prognosis. Patients diagnosed with chronic inflammatory disorders (eg, rheumatoid arthritis (RA), systemic lupus erythematosus (SLE), psoriasis) have an increased risk of coronary heart disease (CHD), stroke, type 2 diabetes (T2DM), peripheral artery disease (PAD), venous thromboembolism (VTE) and CVD-related mortality.^1–6^ Emerging evidence suggests similar associations for ankylosing spondylitis (AS), Crohn’s disease and ulcerative colitis (UC), although this evidence is less consistent.^7 8^ In a prospective primary care cohort, we have documented an increased risk of CVD events associated with diverse chronic inflammatory disorders.^1^ Socioeconomic disadvantage (eg, unemployment) experienced by these patients may underlie disparities in incidence and prevalence of cardiometabolic disorders and are often major barriers to chronic disease prevention and management.^9^ Because such information is less available in patients’ medical records, we have decided to investigate whether our previous study findings from a primary-care context are transferable to a community-based population with a richer sociodemographic data. The aim of the present study was to strengthen the evidence of the original study, to clarify additional issues (eg, anti-inflammatory therapy) and extend its generalisability to other disorders (eg, ankylosing spondylitis) or outcome measures (eg, mortality, PAD, VTE). Given the evidence for increased risk of CVD associated with corticosteroids in the general population,^10^ we hypothesised that the association between inflammatory disorders with cardiometabolic events will be greater among participants treated with corticosteroids or non-steroidal anti-inflammatory drugs (NSAIDs).

## Methods

### Data

The data for the present study come from the UK Biobank, a large population-based prospective study developed to facilitate detailed investigations about the non-genetic and genetic determinants of diseases in middle and old age. The UK Biobank collects detailed phenotype and genotype data from over half a million participants aged 40 to 69 (502 641), including lifestyle, demographics, clinical diagnoses, treatment and genotype information.^11^ There are also plans to incorporate previous medical diagnostic codes, imaging and biomarkers data in the near future. The present study used the baseline data to investigate the cross-sectional association between inflammatory disorders with cardiometabolic risk. In addition, prospective data on mortality was used to investigate the longitudinal association between inflammatory disorders with mortality. For both cross-sectional and prospective investigations, participants reporting a previous diagnosis of RA, SLE, psoriasis, AS, systemic vasculitis, Crohn’s disease and UC disorders represented the exposed group, while those reporting none of these disorders constitute the comparison group. A more detailed description of the UK Biobank data is provided elsewhere.^11^

The UK Biobank has generic ethics approval from the National Health Service National Research Ethics Service (Ref: 11.NW/0382) and all participants provided written informed consent.

### Outcomes

#### Main outcome variables

Self-report of a clinician diagnosis of CHD (ie, myocardial infarction, angina), stroke, T2DM, PAD and VTE events were used to develop the primary outcome measures for the cross-sectional investigation. Traditionally, T2DM is considered an independent risk factor for CVD, and adjusted accordingly. Reflective of the common origins hypothesis,^12^ T2DM and CVDs are gradually operationalised as a multifaceted construct—the cardiometabolic disorder.^13 14^ This framework was adopted in our recent investigation^1^ that documented chronic inflammation as a risk factor for both T2DM and CVD disorders implying a possible role of the inflammatory process in the origin of both CVD and T2DM disorders. Cardiometabolic events are defined using the International Classification of Diseases, edition 10 (ICD-10). To reflect the possibility that inflammatory disorders are associated with diverse cardiometabolic disorders two composite binary measures were developed. A cumulative cardiometabolic measure that classified patients into those with at least one cardiometabolic event (1) versus those without an event (0); and a multiple cardiometabolic measure that grouped participants into those with two or more cardiometabolic events (1) versus those with one or no cardiometabolic event (0).

#### Secondary outcome variables

Official data from the National Health Service’s Centre Registry were used to develop two binary (yes/no) secondary outcomes measures: all-cause mortality and CVD-related mortality.

#### Exposures

The study exposures were represented by seven chronic inflammatory disorders, namely RA, psoriasis, Crohn’s disease, UC, SLE, systemic vasculitis and AS. These measures were based on participants self-report of a clinician diagnosis and were developed into separate binary variables (yes/no). To test the hypothesis that cardiometabolic events and mortality rates may vary with anti-inflammatory therapy and disorder duration, two additional variables were developed for each inflammatory disorder. A therapy variable that used anti-inflammatory prescribing data to classify participants into: no anti-inflammatory therapy, NSAIDs or corticosteroids only therapy, and disease-modifying antirheumatic drugs (DMARDs). An inflammatory disorder duration variable based on the number of years from inflammatory disorder diagnosis to the year of the UK Biobank assessment. This variable grouped participants into tertiles of inflammatory disorder duration.

#### Covariates

Because of the cross-sectional nature of the data at baseline, the study covariates included sociodemographic characteristics, specifically age (continuous measure), gender (female vs male), deprivation, education and ethnicity. Deprivation was based on Townsend deprivation indices derived from aggregated data on car ownership, household overcrowding, owner occupation and unemployment (higher scores represent higher degree of deprivation). For the present study, participants were grouped into quintiles of deprivation. Education (options included degree, Advanced(A) levels/Advanced Subsidiary(AS) levels, O levels/General Certificate of Secondary Education (GCSE), Certificate of Secondary Education (CSEs), National Vocational Qualification (NVQ)/Higher National Diploma (HND)/Higher National Certificate (HNC) or none) was included as a binary variable comparing participants with a degree or professional qualification with those with other qualifications.^15^ Self-reported ethnicity classified participants into white, Asian (eg, India, Pakistan or Bangladesh), Chinese, black, or mixed/other. Lifestyle factors (eg, smoking, physical activity, diet, obesity) are important risk factors for CVD, however, because these were measured at the date of assessment adjusting for them in the analysis may have introduced bias (their value is likely to have changed over time). As educational level is related to lifestyle factors, we adjusted for this factor in the analyses. Two additional binary (yes/no) covariates were included in the sensitivity analyses: self-reported antihypertensive therapy and lipid-lowering therapy. Data on the prevalence of atherogenic risk factors (eg, smoking, body mass index, hypertension, dyslipidaemia) are provided in the online supplementary table S1.

10.1136/heartjnl-2017-311214.supp1 Supplementary material 1

### Statistical methods

#### Cross-sectional study

Descriptive analyses were used to explore differences in demographics characteristics between participants with and without a diagnosis of chronic inflammatory disorder. Multivariable binomial regression analysis was used to estimate the cross-sectional association between each inflammatory disorder with each primary cardiometabolic outcome measure. The same modelling strategy was used to estimate the association between anti-inflammatory therapy and length of exposure. Binomial regression modelling was preferred to logistic regression as it allows an estimation of the relative risk of study outcomes, the preferred estimate when the outcomes’ incidence is common.^16^ The analyses adjusted for age, sex, deprivation, ethnicity and educational level. Sensitivity analyses adjusting (in addition to study covariates) for antihypertensive and lipid-lowering drugs were also performed.

#### Prospective study

Cox proportional hazards regression was used to estimate differences in all-cause and CVD-related mortality rates between participants with and without a diagnosis of chronic inflammatory disorder. These analyses adjusted for age, sex, deprivation, ethnicity and educational level. Participants entered the study at the time of baseline assessment and exited at the time of death or study end. The proportionality assumption was tested using Schoenfeld residuals and was found not to be violated. Sensitivity analyses excluding participants with cardiometabolic disorders at baseline were also performed (data available from the authors).

Within both cross-sectional and prospective studies, random-effects meta-analysis was used to obtain a pooled estimate of the relative risk of primary and secondary outcomes within specific inflammatory disorders. Because adjustment for multiple comparisons could increase the risk of type II errors,^17^ we have interpreted statistical significance within the context of the magnitude of association and previous research findings.^17^ Because less than 0.01% of the data were missing, complete case analyses were performed. All analyses were conducted using STATA V.14, using p <0.5 as the threshold for statistical significance.

## Results

A total of 19 082 (4%) participants with a prior diagnosis of chronic inflammatory disorder have been identified. The most common inflammatory disorders were psoriasis (31%) and RA (28%), followed by Crohn’s disease (13%), UC (7%) ankylosing spondylitis (7%), vasculitis (7%) and SLE (3%) (table 1). The mean age at inflammatory disorder diagnosis ranged from 33 years (psoriasis) to 57 years (systemic vasculitis). A larger proportion of participants diagnosed with inflammatory disorders were in the most deprived quintile relative to the comparison group. With respect to ethnicity, participants of Asian origins reported a threefold increase in the rates of SLE relative to the comparison group.

**Table 1:** Participants characteristics at baseline assessment. Figures are numbers and percentages unless otherwise specified

	RA, n=5764	Psoriasis, n=6286	AS, n=1400	Vasculitis, n=1475	SLE, n=654	UC, n=2659	CD, n=1494	Unexposed, n=483 559
Age—mean (SD)	46 (14)	33 (17)	37 (13)	57 (11)	42 (12)	39 (13)	36 (14)	57 (8)
Gender—Female	4014 (70)	2991 (48)	528 (38)	986 (67)	582 (89)	1427 (54)	861 (58)	261 549 (54)
Ethnicity								
White	5431 (94)	6070 (96)	1352 (97)	1435 (98)	561 (86)	2542 (96)	1451 (98)	453 485 (94)
Mixed	135 (2)	98 (2)	13 (1)	9 (1)	23 (4)	57 (2)	21 (1)	9512 (2)
Asian	73 (1)	16 (0)	4 (0)	8 (0)	42 (6)	16 (1)	9 (1)	7887 (2)
Black	38(1)	31(1)	8 (1)	6 (0)	10 (2)	13 (0)	3 (0)	2850 (1)
Other	54 (1)	41 (1)	13 (1)	10 (1)	13 (2)	23(1)	4 (0)	6019 (1)
Deprivation quintile								
Least deprived	1015 (18)	1181 (19)	268 (19)	321 (22)	115 (18)	539 (20)	274 (18)	96 898 (20)
Second	1011 (18)	1185 (19)	277 (20)	287 (20)	110 (17)	552 (21)	289 (18)	96 292 (20)
Third	1107 (19)	1229 (19)	262 (19)	333 (23)	120 (18)	550 (21)	286 (19)	96 418 (20)
Fourth	1176 (20)	1266 (20)	283 (20)	296 (20)	128 (20)	551 (21)	314 (21)	96 240 (20)
Most deprived	1445 (25)	1420 (23)	309 (22)	235 (16)	180 (28)	464 (17)	327 (22)	95 935 (20)
Qualifications								
Degree level	434 (8)	614 (10)	139 (10)	126 (9)	58 (9)	248 (9)	134 (9)	53 777 (11)

AS, ankylosing spondylitis; CD, Crohn’s disease; RA, rheumatoid arthritis; SLE, systemic lupus erythematosus; UC, ulcerative colitis.

### Cross-sectional results

Table 2 illustrates the distribution of study primary outcome measures within specific inflammatory disorders and the comparison group. The most common combination of specific outcomes and multiple outcome is also reported. Multiple cardiometabolic diseases were more common among SLE (4%) and vasculitis (4%) disorders, and less common among participants reporting Crohn’s disease (2%) or psoriasis (2%) disorders. Participants diagnosed with SLE presented the highest rates of cardiometabolic events, with the exception of T2DM which was more common among participants reporting RA (6%), vasculitis (6%) or psoriasis (6%) disorders. The most common combination of cardiometabolic disorders were CHD with T2DM, CHD with VTE and CHD with stroke.

**Table 2:** Prevalence of individual and most common combination of cardiometabolic outcomes by study inflammatory disorders

	CHD+T2DM	CHD+Stroke	CHD+VTE	CHD+PAD	VTE	T2DM	CHD	Stroke	PAD	Multiple
RA	66 (1)	36 (1)	48 (1)	8 (0)	269 (5)	345 (6)	484 (8)	162 (3)	52 (1)	187(3)
Psoriasis	71 (1)	17(0)	16 (0)	6 (0)	176 (3)	367 (6)	379 (6)	103 (2)	29 (1)	127 (2)
AS	18 (1)	11 (1)	8 (1)	2 (0)	45 (3)	75 (5)	100 (7)	38 (3)	8 (1)	40 (3)
Vasculitis	16 (1)	11 (1)	11 (1)	1 (0)	100 (7)	83 (6)	108 (7)	48 (3)	15 (1)	54 (4)
SLE	2 (0)	9 (1)	11 (2)	4 (1)	83 (13)	27 (4)	52 (8)	39 (6)	25 (4)	33 (4)
UC	26 (1)	7 (0)	21 (1)	2 (0)	148 (6)	142 (5)	172 (6)	49 (2)	15 (1)	70 (3)
CD	8 (1)	7 (1)	6 (0)	0 (0)	69 (5)	53 (4)	62 (4)	23 (2)	12 (1)	25 (2)
Non-exposed	3134 (1)	1325 (0)	1252 (0)	197 (0)	12 131 (3)	19 949 (4)	21 857 (5)	7425 (2)	1350 (0)	6901 (1)

The figures represent the number and percentage (brackets) of participants diagnosed with cardiometabolic events out of the total within each condition or non-exposed group.

AS, ankylosing spondylitis; CD, Crohn’s disease; CHD, coronary heart disease; Multiple, two or more outcome measures (participants from previous columns are also included in this column); PAD, peripheral artery disease; RA, rheumatoid arthritis; SLE, systemic lupus erythematosus; T2DM, type 2 diabetes mellitus; UC, ulcerative colitis; VTE, venous thromboembolism.

Binary regression results revealed increased risk of cardiometabolic diseases (online supplementary table S2) within most inflammatory disorders. The strongest association was revealed with respect to SLE and PAD disorders (relative risk (RR) 17.24, 95% CI 11.36 to 26.19). Participants diagnosed with RA were associated with increased risk of all cardiometabolic outcome measures. The association for other disorders tended to be restricted to specific cardiometabolic disorders.

**Figure 1:**
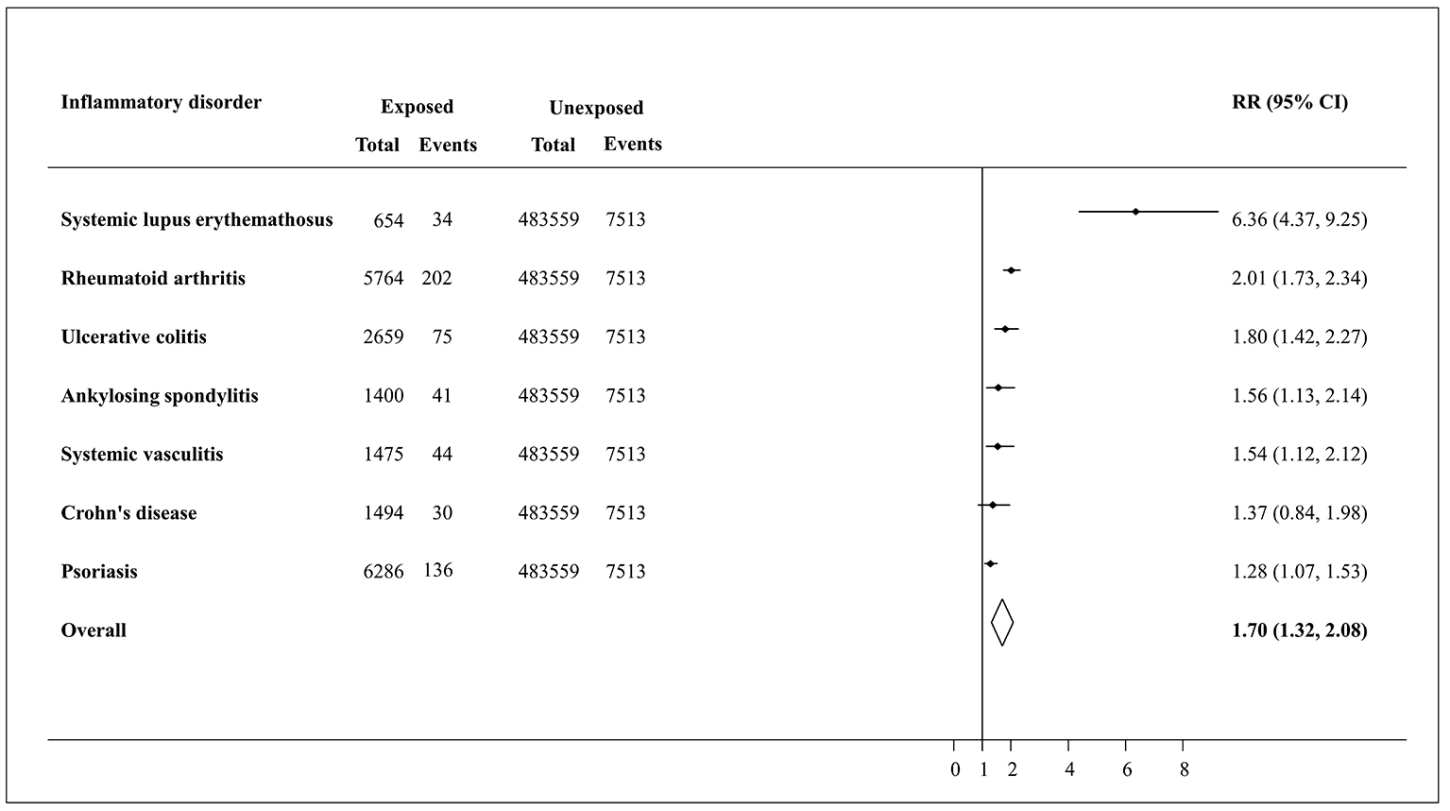
Forest plot displaying random effects meta-analysis of the association between chronic inflammatory disorders with multiple cardiometabolic outcomes. The lines around the dot represent the 95% CI for the effect size. The line drawn perpendicular to the x axis represents the null hypothesis (RR=1). RR, relative risk.

Figure 1 illustrates that participants diagnosed with SLE presented a sixfold adjusted increased risk of multiple (RR 6.36, 95% CI 4.37 to 9.25) cardiometabolic events. The RR for pooled estimate for multiple and cumulative cardiometabolic events was 1.70 (95% CI 1.32 to 2.08). A similar pattern was observed with regards to the cumulative cardiometabolic outcome where the pooled effect size was 1.77 (95% CI 1.45 to 2.10) (online supplementary figure S1).

10.1136/heartjnl-2017-311214.supp2 Supplementary figure 1

**Figure 2:**
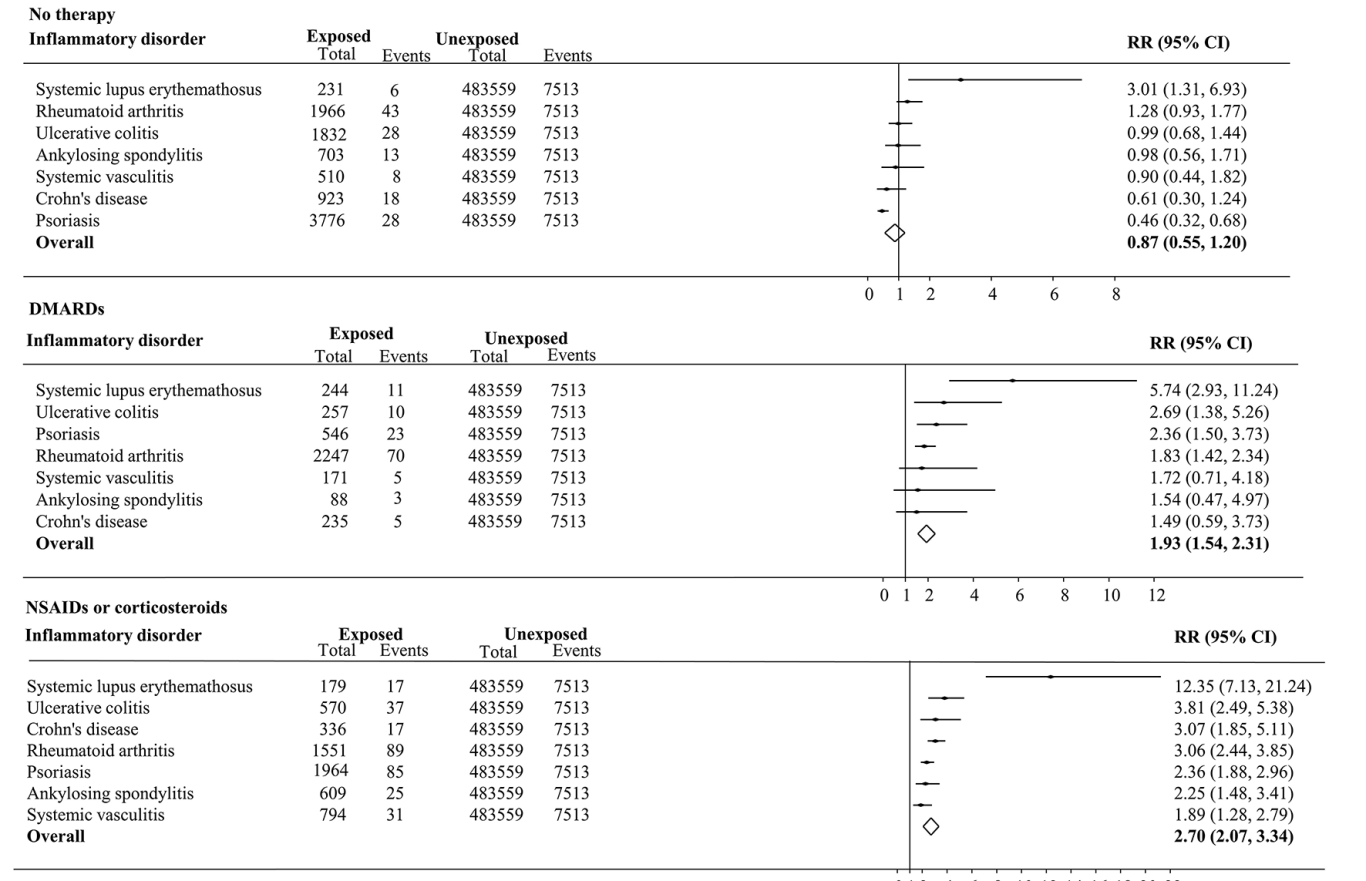
Forest plot displaying random effects meta-analysis for multiple cardiometabolic outcome among participants diagnosed with specific inflammatory disorders who reported no drug therapy, with NSAIDs or corticosteroids only therapy, or with DMARDs therapy compared with those free of inflammatory disorders. RR, relative risk. The line drawn perpendicular to the x axis represents the null hypothesis (RR=1). DMARDs, disease-modifying antirheumatic drugs; NSAIDs, non-steroidal anti-inflammatory drugs.

Figure 2 illustrates that the highest adjusted RR of multiple cardiometabolic events was observed among participants reporting NSAIDs or corticosteroids only therapy. For instance, patients diagnosed with SLE presented a 12-fold adjusted increased risk of multiple cardiometabolic events (RR 12.35, 95% CI 7.13 to 21.24) compared with those without an inflammatory disorder. No statistically significant association was observed among participants without self-reported anti-inflammatory drugs therapy (with the exception of SLE). Similar findings were revealed with regards to the cumulative cardiometabolic outcome events (online supplementary figure S2). Concerning the duration of inflammatory disorders, an increasing dose–response relationship was observed among participants diagnosed with SLE and RA disorders, while the opposite trend was observed among participants diagnosed with psoriasis, AS and UC disorders (online supplementary figures S3 and S4). The findings for individual cardiometabolic outcome are presented in online supplementary figures S3 and S4).

10.1136/heartjnl-2017-311214.supp3 Supplementary figure 2

### Prospective study

**Figure 3:**
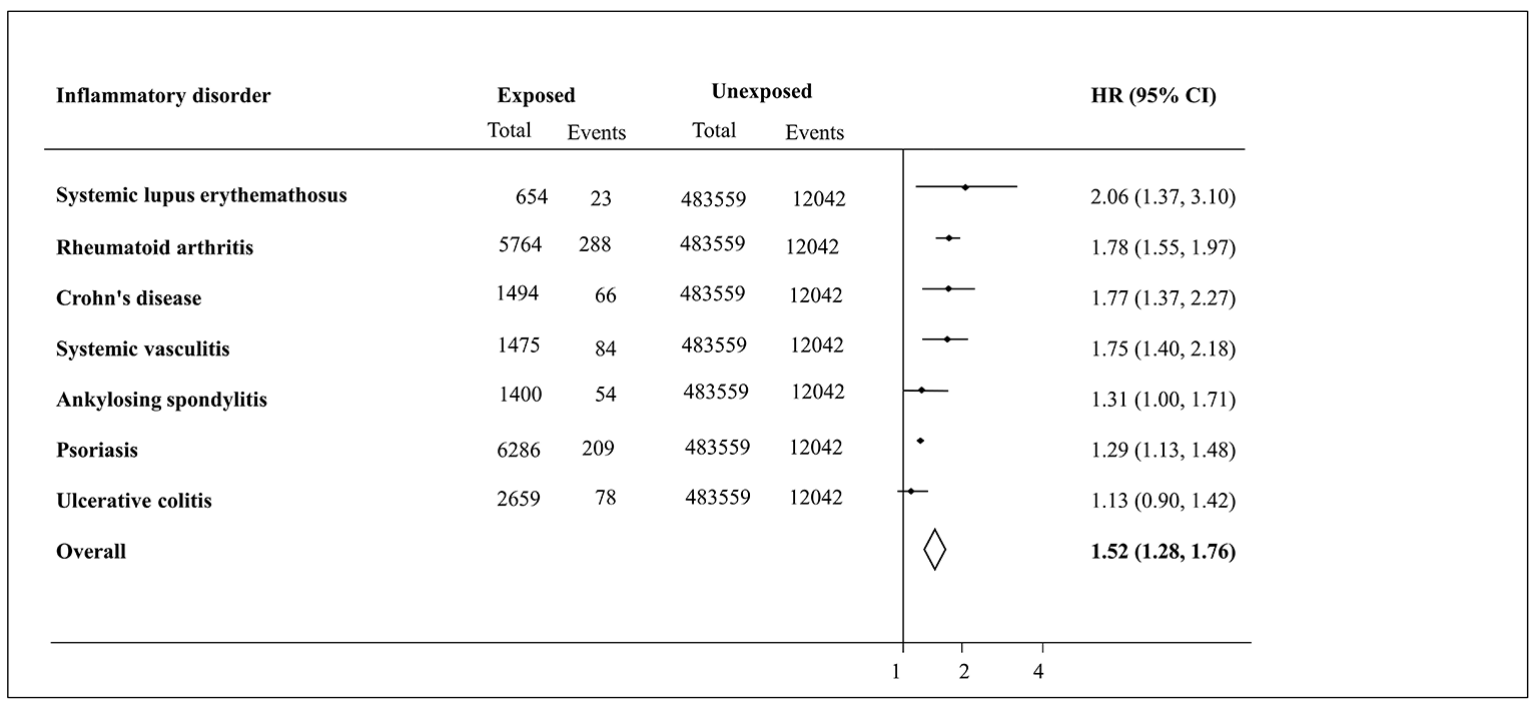
Forest plot displaying random effects meta-analysis for all-cause mortality among participants diagnosed with specific inflammatory disorders compared with those free of inflammatory disorders. The line drawn perpendicular to the x axis represents the null hypothesis (HR=1).

Figure 3 illustrates that participants diagnosed with SLE presented with the highest adjusted HR of all-cause mortality (HR 2.06, 95% CI 1.37 to 3.10) compared with the comparison group. The pooled estimates for all-cause were 1.52 (95% CI 1.28 to 1.76). Similar patterns were observed with regards to CVD-related mortality outcome analysis (online supplementary figure S5).

10.1136/heartjnl-2017-311214.supp6 Supplementary figure 5

### Sensitivity analyses

Sensitivity analyses that also adjusted for antihypertensive and lipid lowering drugs validated the main study findings with regards to cardiometabolic outcomes (online supplementary figure S5). Also, analyses that excluded participants with cardiometabolic events at baseline validated the study findings about mortality outcomes (data not shown).

## Discussion

### Primary outcome measures: cross-sectional study findings

The aim of the present study was to replicate in a community-setting the relationship between specific chronic inflammatory disorders with cardiometabolic outcomes documented in a primary care context, using a similar methodological approach. Adjusting for critical socioeconomic inequalities, the present study findings endorsed the increased risk of cardiometabolic events (eg, CHD, stroke, T2DM) within specific chronic inflammatory disorders and extended these associations to other disorders (eg, AS) and outcome measures (eg, PAD, VTE). A notable finding was that participants diagnosed with pathologically diverse inflammatory disorders were at greater risk of both multiple and cumulative cardiometabolic events compared with their counterparts without inflammatory disorders. The large effect size observed for PAD outcome (specifically within the SLE disorder) relative to other outcome measures (ie, CHD, stroke) indicates a possible strong association, however, the wide confidence intervals questions the precision of the estimates. This association would benefit, thus, from confirmation with larger prospective studies.

Overall, these associations were stronger among participants who were prescribed NSAIDs or corticosteroids, potentially reflecting the increased CVD risk associated with some of these drugs. Participants who were prescribed DMARDs also presented greater rates of cardiometabolic events, but the magnitude of the association was lower. Confounding by indication may explain the findings for DMARDs, given their prescribing indication for patients with more severe underlying inflammation. This suggestion is supported by the evidence of no increased cardiometabolic risk among participants who did not report anti-inflammatory therapies. Patients prescribed DMARDs are likely also to be prescribed NSAIDs or corticosteroids to alleviate disorder-related pain. Thus, the association may be confounded by the increased cardiometabolic risk confer by NSAIDs or corticosteroid drugs.^18^ Certain DMARDs (eg, leflunomide and ciclosporin) have also been linked with increased risk of hypertension,^19^ which may also account for the observed association.

A dose-response association was observed with respect to the duration of inflammatory disorder. Specifically, the risk of cardiometabolic events increased with the duration of RA, SLE, and systemic vasculitis (cumulative outcome) disorders. A reverse trend was apparent, however, within Crohn’s disease, UC and psoriasis (cumulative events) disorders. This reverse trend may be due to an effective disorder management that may obscure a potential association between disorder duration with cardiometabolic risk. There are also suggestions that reduced CVD risk with longer Crohn’s disease duration may be due to progression from an inflammatory to a fibrostenotic disorder phenotype.^20^

The simultaneous investigation of cumulative and multiple cardiometabolic risk within pathologically diverse inflammatory disorders is rarely available.^1 21^ Using a primary-care population, we have recently^1^ identified an increased risk of multiple CVD events within specific inflammatory disorders. The present study documented similar patterns within a community-based population and extended these findings to multiple cardiometabolic outcomes and additional disorders (eg, AS). Previous research associated methotrexate use with lower CVD risk.^22 23^ The present study findings imply increased cardiometabolic risk among DMARDs treated participants in line with Ogdie *et al* findings.^21^ The eclectic definition of DMARDs (eg, methotrexate, ciclosporin, leflunomide, azathioprine) in this study may mask the impact of specific DMARDs on cardiometabolic risk. The study findings are in line with prior studies^24^ suggested a decline in CVD risk associated with increased duration of Crohn’s disease and UC, while the reverse trend was suggested among SLE.^25^

### Secondary outcome measures: prospective findings

With the exception of UC, participants diagnosed with inflammatory disorders presented higher rates of all-cause and CVD-related mortality events relative to inflammatory disorder-free participants. These findings are consistent with prior evidence based on smaller samples.^4 26 27^

## Strengths and limitations

The present study has several strengths including large sample size with detailed information on socioeconomic factors, objectively assessed mortality data, type of chronic inflammatory disorders and cardiometabolic diseases. The study population is representative of the UK population, supporting the generalisability of the findings. As with most observational studies there are several limitations that need discussing. Our analysis for cardiometabolic risk was rather cross-sectional limiting any robust inferences about the temporal association between inflammatory disorders and cardiometabolic risk. However, the direction of the association and effect sizes were similar to previous prospective studies.^1^ Cardiometabolic and inflammatory disorders events were identified via self-reports of a clinician diagnosis, increasing the risk of ascertainment bias but unlikely to introduce a systematic error.^28^ Possible decreased specificity of self-reported diagnoses may have led to overestimation of the reported rates of CVD events, especially among patients with chronic inflammatory disorders. This is particularly true for immune mediated inflammatory disorders (eg, SLE and RA) where common conditions such as pericarditis, myocarditis and pleuritis can mimic CHD; cerebritis can mimic stroke; and peripheral neuropathy may mimic peripheral vascular disease (PVD). Recent evidence^29^ documented, however, high validity and agreement rates between self-reported cardiometabolic events with medical records among patients diagnosed with RA. The prevalence rates of inflammatory disorders in this study are also in line with evidence from clinical settings.^21 27^ Also, the study findings are consistent with earlier results based on a large prospective study^1^ with primary care patients using physicians recorded diagnoses. Future plans to link the UK Biobank data with participants’ medical care records will provide the opportunity to validate the reliability of clinical diagnoses recording in the UK Biobank data. The study population was mainly white European with other ethnic groups being under-represented, questioning the generalisability of the study findings to non-white European populations. The number of outcome events across the different combinations of inflammation disorders was relatively low, which may account for the apparent lack of statistical significance in some conditions (eg, Crohn’s disease). Finally, despite the large sample size available for analysis, the smaller number of cardiometabolic events (eg, PAD) may have inflated the magnitude of the observed association within some inflammatory disorders (eg, SLE), although unlikely to alter the direction of association or statistical significance. The prospective nature of the UK Biobank study will ensure increased incidence of cardiometabolic events providing for more precise estimates in future studies.

## Conclusion

Compared with the general population, adults diagnosed with clinically diverse inflammatory disorders present heightened rates of multiple cardiometabolic diseases. This risk varied with anti-inflammatory therapy and time of duration of the disorder. For some inflammatory disorders, the increased risk was detectable early in the course of disorder, supporting the public health value of early screening and effective intervention strategy. Recent evidence from primary care data^30^ suggests that we are falling short of this suggestion. The findings of increased cardiometabolic risks associated with NSAIDs and/or corticosteroids drugs reinforce the need for a more cautionary approach to the prescription of these drugs. The magnitude of the association for SLE supports the development of clinical recommendations for early screening and regular monitoring of cardiometabolic risk in these patients, similar to RA and psoriasis subgroups. Also, the evidence that cardiometabolic risk varied with anti-inflammatory therapy, endorses future evaluations into the potential role of specific DMARDs (alone or in combination with vascular risk therapies (ie, statins)) into the onset and prognosis of cardiometabolic events within specific inflammatory disorder. The UK Biobank follow-up is currently being conducted via linkage with electronic medical records that will, in due course, allow more robust evaluations of the history and treatment of specific inflammatory disorders, and how these factors influence the development and progression of specific and multiple cardiometabolic diseases. In addition, the availability of genotype and imaging data will provide improved opportunities for risk stratification and predictive biomarkers for early cardiometabolic risk identification.

**Key messages**

**What is already known on this subject?**

Previously we have identified a 20% overall increment in the risk of multiple cardiometabolic disorders among patients diagnosed with chronic inflammatory disorders in primary care settings. The generalisability of this finding to community-based populations is, however, uncertain.

**What might this study add?**

In a large community-based population, a 70% overall increment in the risk of multiple cardiometabolic disorders associated with chronic inflammation was observed. The overall risk of multiple cardiometabolic events was almost three times (relative risk=2.72) greater among participants prescribed non-steroidal anti-inflammatory drugs compared with those without a chronic inflammation diagnosis. Also, a 52% overall increased risk of all-cause mortality was observed among participants diagnosed with chronic inflammation relative to those without a chronic inflammation.

**How might this impact on clinical practice?**

The study findings confirm that inflammatory disorders increase risk of multiple cardiovascular events, similar to the risk conferred by diabetes mellitus and chronic kidney disease. This evidence endorses the development of specific clinical guidelines to facilitate cardiovascular disease risk prevention across diverse inflammatory disorders.

10.1136/heartjnl-2017-311214.supp4 Supplementary figure 3

10.1136/heartjnl-2017-311214.supp5 Supplementary figure 4
