# Supplementary material for: Cardiovascular and type 2 diabetes morbidity and all-cause mortality among diverse chronic inflammatory disorders
Source: Heart. 2017 Jun 10;103(23):1867–73. doi: 10.1136/heartjnl-2017-311214 (PMC5749371; doi:10.1136/heartjnl-2017-311214)
Supplement: Supplementary material 1 [file heartjnl-2017-311214supp001.pdf]

Table S1 Prevalence of vascular risk factors by inflammatory disorder. Figures are numbers and percentages unless otherwise specified.

	RA	Psoriasis	AS	Vasculitis	SLE	UC	CD	Unexposed
Hypertension	2080(36)	1965(31)	494(35)	580(39)	220(34)	716(27)	389(26)	128968(27)
Hypercholesterolemia	952(17)	912(15)	230(16)	248(17)	76(12)	305(11)	121(8)	60911(13)
Obese (BMI≥30)	1823(32)	1886(30)	350(25)	422(29)	168(26)	581(22)	276(19)	116807(24)
Smoking								
Current	719(13)	953(15)	198(14)	123(8)	78(12)	168(6)	231(16)	50,483(11)
Ex-smoker	2317(41)	2566(41)	564(41)	558(38)	229(35)	1176(44)	601(40)	165002(34)
Never	2679(46)	2741(44)	627(45)	787(54)	344(53)	1304(49)	656(44)	264114(55)

Note: RA=Rheumatoid arthritis, AS=Ankylosing spondylitis, SLE=Systemic lupus erythemathosus, UC=Ulcerative colitis, CD=Crohn's disease. M=mean, sd=standard deviation. BMI – body mass index, kg/m^2.

Table S2 Adjusted[1] Risk ratios and associated 95% Confidence intervals for the association between inflammatory disorders and cardiometabolic risk

	CHD	Stroke	T2DM	VTE	PAD
Rheumatoid arthritis	1.72(1.56-1.91)	1.56(1.32-1.83)	1.41(1.09-1.82)	1.60(1.41-1.81)	3.10(2.34-4.11)
Psoriasis	1.26(1.13-1.41)	1.03(0.84-1.25)	1.56(1.23-1.98)	1.12(0.96-1.31)	1.60(1.10-2.33)
Ankylosing spondylytis	1.29(1.04-1.61)	1.54(1.11-2.14)	0.90(0.48-1.68)	1.28(0.95-1.73)	1.91(0.95-3.85)
Systemic vasculitis	1.30(1.05-1.60)	1.74(1.30-2.33)	1.14(0.67-1.94)	2.19 (1.78-2.69)	3.09(1.82-5.26)
SLE	3.23(2.37-4.40)	5.34(3.82-7.45)	1.93(0.91-4.10)	5.73(4.52-7.27)	17.24(11.36-26.19)
Ulcerative colitis	1.40(1.19-1.65)	1.15(0.86-1.53)	1.97(1.43-2.72)	2.23(1.88-2.64)	2.05(1.23-3.42)
Crohn's disease	0.93(0.72-1.22)	1.03(0.68-1.56)	1.31(0.75-2.26)	1.85(1.44-2.37)	3.01(1.70-5.34)
**Nonexposed**	**Reference**	**Reference**	**Reference**	**Reference**	**Reference**

Note: SLE=Systemic lupus erythemathosus, CHD=coronary heart disease, VTE=venous thromboembolism, T2DM- type 2 diabetes mellitus, PAD=peripheral artery disease. [1] The analyses were adjusted for age, gender, ethnicity, deprivation index, and educational level. The adjusted risk ratios represent the unique relationship between each inflammatory condition with each outcome measure (ie the models for rheumatoid arthritis exclude patients diagnosed with psoriasis, ankylosing spondylitis, systemic vasculitis, SLE, ulcerative colitis, and Crohn's disease.

Table S3 Adjusted[1] Risk ratios and associated 95% Confidence intervals for the association between inflammatory disorders and cardiometabolic outcome measures by the type of drug therapy.

	CHD	Stroke	T2DM	VTE	PAD
Rheumatoid arthritis					
No drugs	1.00(0.80-1.25)	1.05(0.74-1.47)	1.26(0.79-2.01)	1.35(1.07-1.70)	1.45(0.72-2.91)
NSAIDs[a]	2.57(2.18-3.04)	2.37(1.84-3.04)	1.74(1.13-2.66)	1.69(1.34-2.13)	4.50(2.90-6.97)
DMARDs	1.75(1.49-2.06)	1.40(1.07-1.84)	1.29(0.85-1.96)	1.74(1.44-2.10)	3.50(2.31-5.31)
Psoriasis					
No drugs	0.47(0.38-0.59)	0.76(0.56-1.03)	1.08(0.74-1.57)	0.99(0.80-1.23)	1.28(0.74-2.22)
NSAIDs[a]	2.47(2.13-2.86)	1.40(1.04-1.87)	1.86(1.29-2.70)	1.20(0.92-1.55)	2.42(1.43-4.11)
DMARDs	2.39(1.77-3.22)	1.33(0.75-2.38)	3.54(2.06-6.06)	1.71(1.13-2.58)	0.63(0.09-4.47)
Ankylosing spondylytis					
No drugs	0.66(0.44-0.98)	1.07(0.61-1.86)	0.89(0.37-2.16)	1.17(0.76-1.81)	1.92(0.72-5.14)
NSAIDs[a]	2.12(1.59-2.81)	2.27(1.49-3.46)	1.05(0.43-2.53)	1.34(0.86-2.10)	1.67(0.53-5.24)
DMARDs	1.36(0.62-2.96)	0.57(0.08-4.12)	-	1.72(0.63-4.71)	3.31(0.45-24.26)
Systemic vasculitis					
No drugs	0.58(0.35-0.96)	1.00(0.52-1.94)	1.23(0.50-2.99)	1.65 (1.10-2.49)	2.63(0.98-7.07)
NSAIDs[a]	1.63(1.26-2.12)	2.06(1.43-2.96)	1.17(0.58-2.36)	2.41(1.85-3.15)	2.38(1.06-5.34)

DMARDs	1.93(1.12-3.32)	2.38(1.11-5.12)	0.73(0.10-5.22)	2.72(1.54-4.80)	8.15(2.99-22.25)
SLE					
No drugs	1.84(0.97-3.46)	2.15(0.95-4.90)	2.35(0.74-7.40)	4.62(3.01-7.11)	13.35(6.27-28.46)
NSAIDs[a]	5.99(3.77-5.91)	10.11(6.22-16.44)	2.00(0.49-8.13)	5.10(3.18-8.18)	18.41(8.57-39.57)
DMARDs	2.80(1.60-4.91)	5.30(3.02-9.30)	1.49(0.37-6.08)	7.46(5.25-10.62)	20.52(10.72-39.28)
Ulcerative colitis					
No drugs	0.69(0.53-0.90)	0.88(0.60-1.32)	1.70(1.12-2.60)	1.97(1.59-2.44)	1.00(0.42-2.42)
NSAIDs[a]	3.44(2.69-4.40)	1.52(0.91-2.56)	2.63(1.47-4.69)	2.92(2.13-4.01)	4.86(2.40-9.82)
DMARDs	2.20(1.38-3.50)	2.12(1.04-4.31)	2.22(0.82-6.00)	2.53(1.49-4.30)	2.92(0.72-11.84)
Crohns disease					
No drugs	0.36(0.21-0.60)	0.40(0.15-0.89)	0.84(0.35-2.04)	1.70(1.23-3.26)	1.66(0.62-4.46)
NSAIDs[a]	2.23(1.55-3.22)	1.81(0.96-3.41)	2.41(1.07-5.46)	2.52(1.61-3.94)	6.32(2.81-14.23)
DMARDs	1.25(0.67-2.35)	2.40(1.17-4.91)	1.30(0.32-5.20)	1.43(0.70-2.91)	3.19(0.79-12.92)

Note: SLE=Systemic lupus erythemathosus, CHD=coronary heart disease, VTE=venous thromboembolism, T2DM- type 2 diabetes mellitus, PAD=peripheral artery disease. NSAIDs= non-steroidal anti-inflammatory drugs. DMARDs= disease modifying anti-rheumatic drugs. [a] – and/or corticosteroids drugs. The analyses were adjusted for age, gender, ethnicity, deprivation index, and educational level.

Table S4 Adjusted[1] Risk ratios and associated 95% Confidence intervals for the association between inflammatory disorders and cardiometabolic outcome measures by the time of duration of each inflammatory disorder

	CHD	Stroke	T2DM	VTE	PAD
Rheumatoid arthritis					
1st	1.66(1.40-1.98)	1.31(0.98-1.76)	1.33(0.86-2.05)	1.20(0.94-1.52)	2.31(1.36-3.93)
2nd	1.62(1.35-1.95)	1.63(1.23-2.16)	1.42(0.91-2.22)	1.78(1.44-2.20)	3.22(1.99-5.22)
3rd	1.90(1.60-2.25)	1.76(1.35-2.31)	1.48(0.96-2.29)	1.86(1.52-2.27)	3.87(2.51-5.98)
Psoriasis					
1st	1.33(1.10-1.60)	1.13(0.82-1.57)	2.24(1.59-3.15)	1.27(0.99-1.63)	1.51(0.78-2.92)
2nd	1.38(1.14-1.66)	1.08(0.76-1.53)	1.38(0.88-2.15)	1.33(1.03-1.71)	1.82(0.97-3.40)
3rd	1.10(0.91-1.31)	0.88(0.62-1.26)	1.08(0.67-1.74)	0.82(0.61-1.10)	1.48(0.77-2.86)
Ankylosing spondylytis					
1st	1.44(0.98-2.13)	1.31(0.69-2.48)	1.75(0.78-3.95)	1.34(0.81-2.22)	2.24(0.71-7.03)
2nd	1.23(0.84-1.80)	1.80(1.06-3.08)	0.28(0.04-1.98)	1.40(0.85-2.31)	2.88(1.08-7.73)
3rd	1.23(0.86-1.76)	1.50(0.86-2.61)	0.73(0.23-2.27)	1.10(0.63-1.91)	0.69(0.10-4.91)
Systemic vasculitis					
1st	1.56(1.15-2.10)	1.41(0.86-2.33)	1.20(0.53-2.69)	2.04(1.46-2.86)	3.23(1.44-7.25)
2nd	0.97(0.64-1.49)	1.38(0.78-2.45)	0.25(0.04-1.79)	1.90(1.28-2.81	4.24(1.88-9.55)

3rd	1.31(0.87-1.97)	2.70(1.70-4.30)	2.14(1.00-4.55)	2.74(1.92-3.98)	1.60(0.40-6.43)
SLE					
1st	3.62(2.19-5.98)	3.24(1.59-6.64)	2.53(0.80-8.02)	3.09(1.83-5.20)	17.17(8.41-35.05)
2nd	2.63(1.28-4.33)	6.27(3.64-10.83)	-	7.34(5.01-10.76)	15.10(7.02-32.46)
3rd	3.76(2.25-6.30)	6.55(3.87-11.07)	3.30(1.21-9.01)	7.10(4.87-10.34)	19.47(9.87-38.43)
Ulcerative colitis					
1st	1.64(1.26-2.14)	1.64(1.07-2.51)	1.58(0.84-2.96)	1.98(1.45-2.70)	2.08(0.86-5.02)
2nd	1.32(0.97-1.79)	1.28(0.79-2.10)	2.23(1.29-3.87)	2.15(1.58-2.92)	2.20(0.91-5.32)
3rd	1.27(0.97-1.67)	0.63(0.34-1.17)	2.11(1.26-3.52)	2.51(1.93-3.27)	1.89(0.78-4.57)
Crohns disease					
1st	1.29(0.86-1.92)	1.15(0.60-2.24)	1.17(0.44-3.13)	1.97(1.30-2.99)	5.91(2.92-11.97)
2nd	0.67(0.38-1.18)	1.04(0.49-2.20)	0.98(0.31-3.05)	1.97(1.29-3.02)	1.59(0.40-3.68)
3rd	0.83(0.52-1.33)	0.89(0.42-1.90)	1.73(0.77-3.90)	1.63(1.05-2.53)	1.46(0.36-5.86)

Note: SLE=Systemic lupus erythemathosus, CHD=coronary heart disease, VTE=venous thromboembolism, T2DM- type 2 diabetes mellitus, PAD=peripheral artery disease. The analyses were adjusted for age, gender, ethnicity, deprivation index, and educational level.

Table S5 Adjusted[1] Risk ratios and associated 95% Confidence intervals for the association between inflammatory disorders and cardiometabolic disease risk after adding antihypertensive and lipid lowering therapy to the adjusted estimation models

	CHD	Stroke	T2DM	VTE	PAD	Multimorbidity	Cumulative
Rheumatoid arthritis	1.62(1.45-1.81)	1.40(1.19-1.66)	1.24(0.95-1.60)	1.56(1.38-1.77)	2.91(2.20-3.86)	1.83(1.56-2.15)	1.62(1.49-1.76)
Psoriasis	1.24(1.09-1.40)	0.98(0.80-1.20)	1.49(1.17-1.90)	1.11(0.95-1.29)	1.51(1.03-2.20)	1.22(1.01-1.47)	1.23(1.12-1.34)
Ankylosing spondylytis	1.23(0.96-1.57)	1.45(1.03-2.04)	0.82(0.44-1.55)	1.26(0.94-1.70)	1.85(0.92-3.73)	1.45(1.03-2.04)	1.24(1.03-1.49)
Systemic vasculitis	1.32(1.04-1.67)	1.70(1.25-2.30)	1.07(0.63-1.82)	2.17(1.76-2.67)	3.04(1.79-5.18)	1.55(1.11-2.16)	1.70(1.44-2.00)
SLE	2.86(2.01-4.05)	4.64(3.26-6.60)	1.49(0.69-3.22)	5.55(4.38-7.04)	15.57(10.19-23.79)	5.63(3.70-8.55)	5.67(4.58-7.01)
Ulcerative colitis	1.56(1.31-1.86)	1.18(0.88-1.58)	2.06(1.47-2.87)	2.24(1.89-2.65)	2.09(1.25-3.49)	1.98(1.55-2.54)	1.88(1.67-2.13)
Crohn’s disease	1.08(0.82-1.45)	1.11(0.73-1.69)	1.44(0.82-2.09)	1.87(1.46-2.39)	3..34(1.77-5.56)	1.65(1.12-2.42)	1.43(1.19-1.73)
**Nonexposed**	**Reference**	**Reference**	**Reference**	**Reference**	**Reference**	**Reference**	**Reference**

Note: SLE=Systemic lupus erythemathosus, CHD=coronary heart disease, VTE=venous thromboembolism, T2DM- type 2 diabetes mellitus, PAD=peripheral artery disease. [1] The analyses were adjusted for age, gender, ethnicity, deprivation index, educational level, antihypertensive medications, and lipid lowering medications.

Figure S1 Forest plot displaying random effects meta-analysis of the association between chronic inflammatory disorders with cumulative cardiometabolic outcomes. RR- relative risk; CI- confidence interval. The lines around the dot represent the 95% confidence interval for the effect size. The line drawn perpendicular to the X axis represents the null hypothesis (RR=1).

Figure S2 Forest plot displaying random effects meta-analysis for cumulative cardiometabolic outcome among participants diagnosed with specific inflammatory disorders who reported no drug therapy, with NSAIDs or corticosteroids only therapy, or with DMARDs therapy compared to those free of inflammatory disorders. RR- relative risk; CI- confidence interval. The line drawn perpendicular to the X axis represents the null hypothesis (RR=1). DMARD = disease modifying anti-rheumatic drugs; NSAIDs = non-steroidal anti-inflammatory drugs.

Figure S3 Forest plot displaying random effects meta-analysis for multiple cardiometabolic outcome associated with the tertiles of disorder duration relative to those free of inflammatory disorders. RR- relative risk; CI- confidence interval. The line drawn perpendicular to the X axis represents the null hypothesis (RR=1). DMARD = disease modifying anti-rheumatic drugs; NSAIDs = non-steroidal anti-inflammatory drugs.

Figure S4 Forest plot displaying random effects meta-analysis for cumulative cardiometabolic outcome associated with the tertiles of disorder duration to those free of inflammatory disorders. RR- relative risk; CI- confidence interval. The line drawn perpendicular to the X axis represents the null hypothesis (RR=1). DMARD = disease modifying anti-rheumatic drugs; NSAIDs = non-steroidal anti-inflammatory drugs.

Figure S5 Forest plot displaying random effects meta-analysis for CVD-related mortality among participants diagnosed with specific inflammatory disorders compared to those free of inflammatory disorders. HR- hazard ratio; CI- confidence interval. The line drawn perpendicular to the X axis represents the null hypothesis (RR=1).
