# Supplementary figures and images for: Cardiovascular and type 2 diabetes morbidity and all-cause mortality among diverse chronic inflammatory disorders
Source: Heart. 2017 Jun 10;103(23):1867–73. doi: 10.1136/heartjnl-2017-311214 (PMC5749371; doi:10.1136/heartjnl-2017-311214)

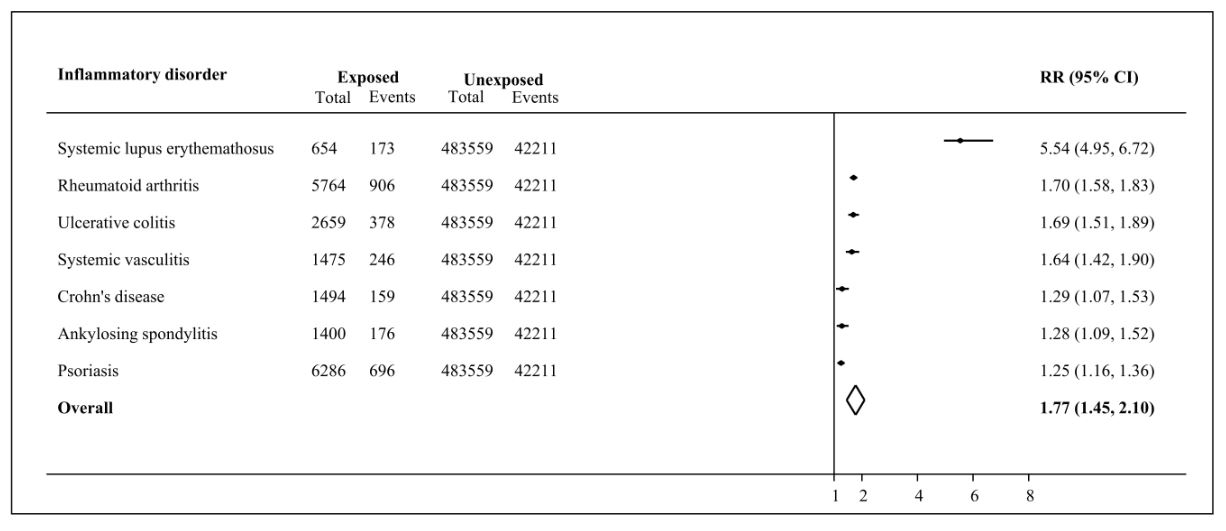

Supplement: Supplementary figure 1 [file heartjnl-2017-311214supp002.jpg]

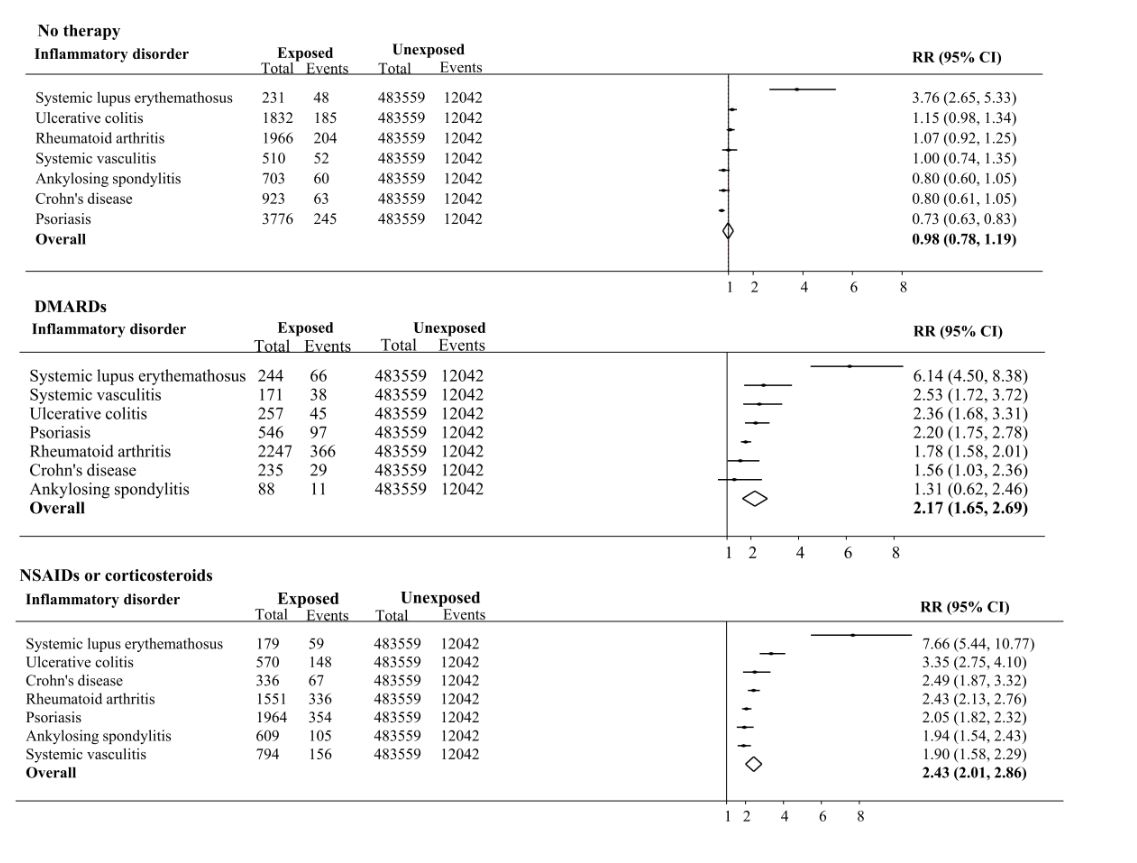

Supplement: Supplementary figure 2 [file heartjnl-2017-311214supp003.jpg]

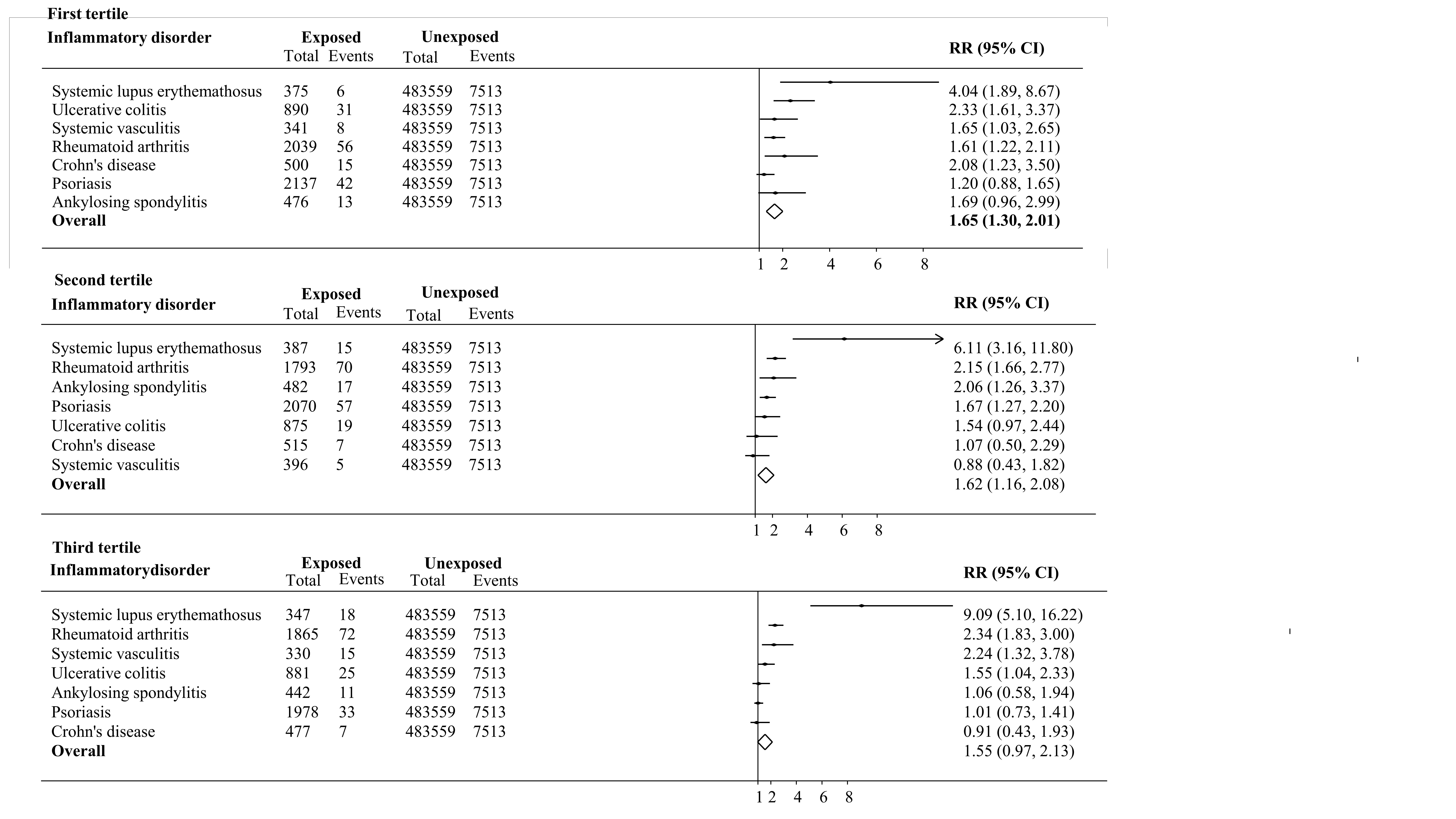

Supplement: Supplementary figure 5 [file heartjnl-2017-311214supp004.jpg]

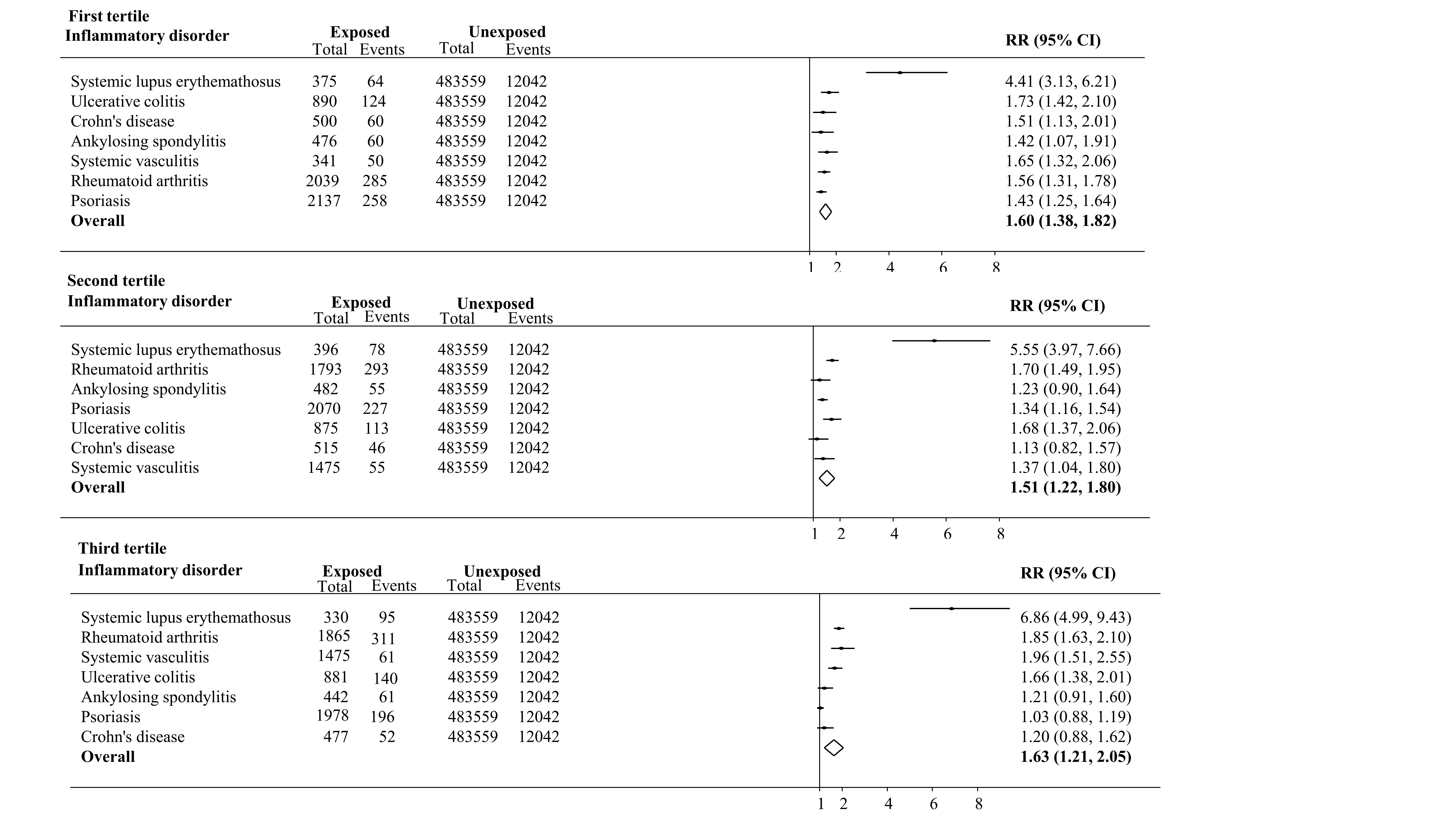

Supplement: Supplementary figure 3 [file heartjnl-2017-311214supp005.jpg]

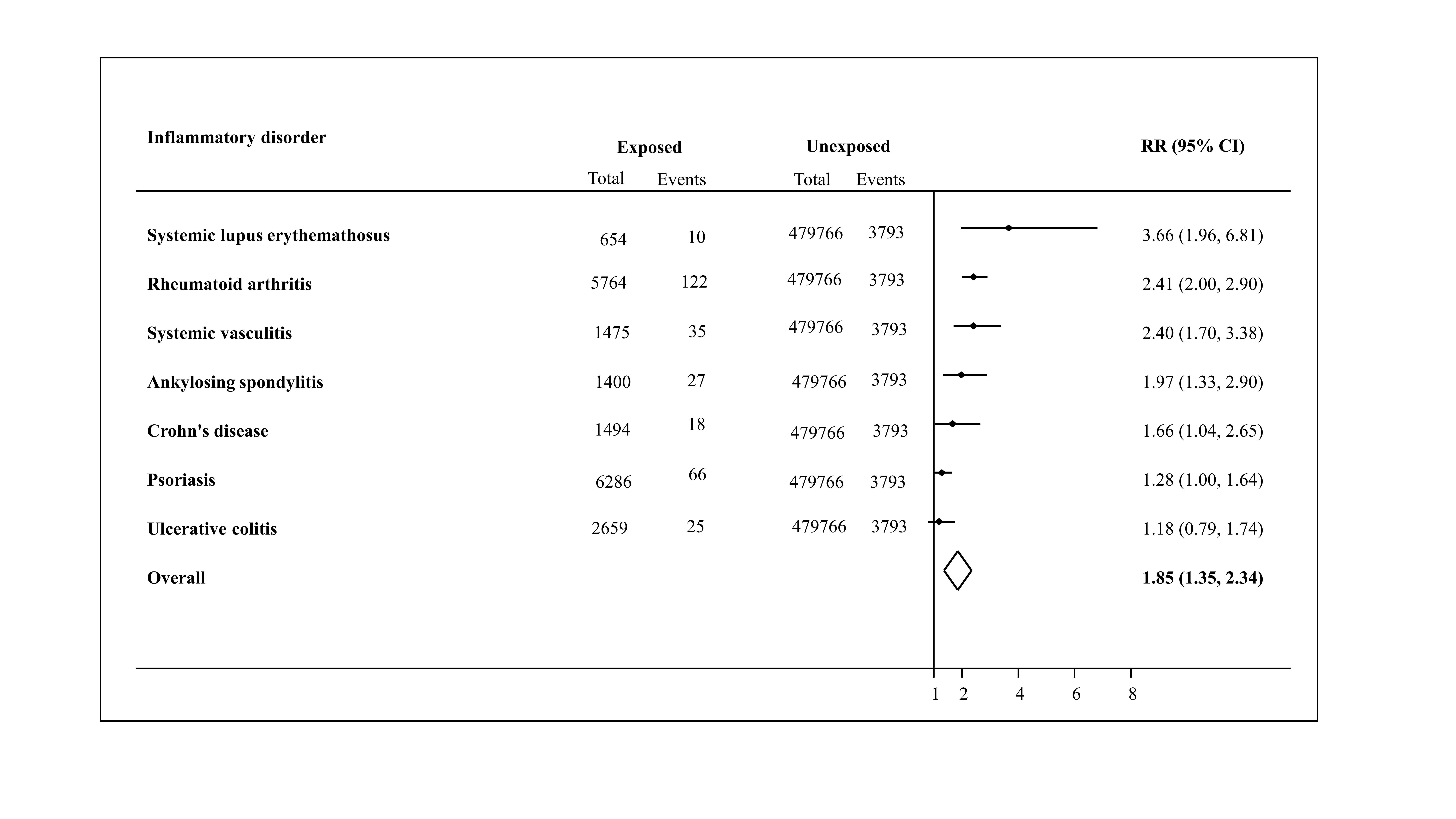

Supplement: Supplementary figure 4 [file heartjnl-2017-311214supp006.jpg]
